# An Account of an Epidemic of Diphtheria

**Published:** 1860-04

**Authors:** Wm. L. Wells

**Affiliations:** Milwaukee, Wisconsin


THE CHICAGO
MEDICAL EXAMINER

VOL. I. APRIL, 1860. No. 4

### ORIGINAL COMMUNICATIONS.

### AN ACCOUNT OF AN EPIDEMIC OF DIPHTHERIA.

By WM. L. WELLS, M. D.. of Milwaukee, Wisconsin. *

This epidemic occurred during the months of July, August, September, and October, in a farming country in the vicinity of the city of Milwaukee. The extent of country over which it prevailed occupies about three square miles, which includes parts of the townships of East and West Granville and Menomonee, containing a population approximating 800 souls. The country is pleasingly undulating, and equally divided between cultivated and timber land. It is watered by two small rivers, and on many of the farms there are springs, with smaller streams flowing from them. The soil is of the lime-stone formation, with a loose sub-soil favorable to the easy penetration of water. There having been but little rain during the spring and summer, the rivers were lower than usual, and many of the smaller streams had completely disappeared. The wet meadows, and one or two swamps in the affected locality, usually supplied with water from springs, were dry. As for the climate of this region and neighborhood, we will simply state, that the summers are short and cool, vegetation is never exposed to a burning sun, so favorable, according to some theorists, to the creation of epidemic causes. The winters are long and cold, but the atmosphere is dry; these facts giving to Wisconsin the universal reputation of being one of the most healthy regions in the states. The townships which suffered from this epidemic, have been free from epidemics for several years. Scarlet fevers prevailed to some extent, and with more

* This epidemic occurred in the practice of Dr. S. S. Clark, of Menomonee. Having a large circuit to ride, and being unable to attend to this sudden increase of practice, he requested my aid. Thus was I enabled to watch the progress of the epidemic. I am indebted to him, also, for his assistance in this article, as I have gathered many facts from his notes and cases.

than their ordinary mortality five years ago. The diseases of this neighborhood have been such as are incident to any similar community, with the exception, perhaps, of occasional seasons when malarious influences would stamp the character of prevailing diseases. The year preceding the epidemic was a remarkably healthy one, and less malarious diseases than usual, though these have been steadily declining for some years.

This district is almost entirely settled by Pennsylvania Germans, well off in worldly goods. They live in well built and comfortable farm houses, and compared with foreign settlers are more cleanly in their household arrangements, and pay stricter attention to domestic hygiene. The adults are strong and robust, the children generally fine looking and healthy.

This epidemic, as we have already said, commenced in July, and ended in the latter part of October. It seemed to have arrived at the height of its violence about the middle of August, it then abated, and in September it appeared to have entirely ceased; but in October it broke out again with increased severity, thirty-five being seized in the short space of three days. The morbific agency ceased to act as suddenly as it began the second time, and in November it had entirely disappeared.

There were 133 persons attacked, 26 adults and 107 children. The majority of cases were between the ages of 2 and 10. Of the adults there was one aged 63. There were but four deaths, all occurring in children aged 3, 5, 8 and 10. Three of the fatal cases occurred in the same family, and were the first attacked; the fourth was the first case after the interval of repose of the epidemic influence. Two died from a rapid and extreme prostration; in one of these the pharyngial inflammation terminated in gangrene; one died three weeks after all traces of the local affection had disappeared, suffering at the time with intense anæmia and paralysis of the lower extremities, and the fourth died with croupal symptoms. In describing this epidemic, we will simply give its general features, as individual cases would not furnish sufficient variety to be interesting. Those cases, however, which illustrate certain special phenomena bearing upon disputed points in diagnosis and pathology, will be mentioned.

The symptoms of this disease have been so faithfully given by Bretonneau, that his description would suit the characteristics of epidemics as witnessed in France, but that his observations did not take in the whole disease is evident from what subsequent observers have accomplished. The general law which governed epidemics of his day are the same to day, though there have been variations in its expression, doubtless dependent upon local influences, such as geographical position, climate, etc., and it is from the sum of these differences, so to speak, that we can arrive at any true conclusion as to its nature. Owing to a loose and extended nomenclature of this disease, observers have fallen into errors, some denying its specific character, others claiming its identity with other diseases, others still, that it is a disease *suigeneris*; and the consequent confusion in their descriptions of epidemics, not only detracts from the value of their observations, but it impedes progress in the investigations of others. Careful and faithful records of different epidemics can alone bring order out of this confusion. By a close attention to the symptoms are we made acquainted with the laws of the disease, and thus will be enabled to distinguish it by a name which can never be misapplied. We shall, therefore, confine ourselves to those phenomena as they appeared to us during this epidemic, saving such reflections and conclusions arrived at for that part of our article under the head of remarks.

*Symptoms.*—There was no uniformity in its manner of access. The prodromata, using the term in its usual signification, those symptoms existing before the formation of the false membrane, when they exist were from one to several days duration. These though common at the onset of many diseases, should arouse the suspicion of the practitioner when he meets with them during the prevalence of an epidemic of diphtheria. These are, a chill, headache, a febrile condition, hot skin, tongue coated, loss of appetite, nausea, vomiting, a feeling of malaise, a slight soreness of the throat, some difficulty in deglutition, and on examination of the fauces a more or less redness of the mucous membrane. These symptoms varied in their appearance and intensity in different cases; in some they were so slight as to attract but little attention; in others they were so severe as to confine the patient to bed for several days before the appearance of the false membrane. In several instances they seemed to constitute the whole disease; repeated and most careful examinations of the fauces and cutaneous surface revealed no membranous exudation. Again, the false membrane was in some cases the first indication of the disease.

*Symptoms referable to the Throat.*—In some cases pain was complaned of only during the act of deglutition, in others it was constant and severe, but this symptom bore no relation to the severity of the local affection. The mucous membrane more or less congested, but in no instance could we discover the peculiar ecchymotic and striated appearance described by Bretonneau and other authors. The tonsils in some cases were tumefied, so much so at times as to form a serious complication; any considerable degree of tumefaction however was an exception. The adventitious membrane generally made its appearance on the second or third day—in a few instances as late as fifth or sixth day—and this if not checked would rapidly spread; in but one instance did it pass to the air passages producing croupal symptoms, the case proved fatal.

*False Membrane.*—This would vary in its locality of first appearance, usually however it was first discovered on one of the tonsils, rarely on both at the same time. In some it commenced by small white spots which would soon unite forming a continuous sheet, in others a patch of size would be developed in a few hours. Once formed, its tendency was to spread, and unless arrested, one or both tonsils with their pillars and the soft palate would be invaded. The pharynx was its exceptional seat. Occasionally it was seen in and about the external openings of the nares, but in most of these cases there existed a herpes or eczema. Its color and thickness would vary in different cases, and according to the length of time it remained undisturbed upon the mucous membrane. Sometimes it was white, resembling a thin layer of mucus, at other times it was yellow, which, on a congested and perhaps tumefied surface, put on the appearance of an ulcer. Again it was gray or ash-colored, which with a fœted oder might be mistaken for commencing gangrene. The false membrane was more or less adherent to the subjacent mucous surface; at times it was easily detached, leaving the mucous membrane in its integrity; at others its removal would be followed by some bleeding, revealing a slight erosion. Once detached it was soon reproduced, and this removal and reproduction would in many cases be repeated for several days before it disappeared, which took place as early as the second and as late as the fourteenth day. The peculiar characteristics of this membrane have received such minute descriptions from authors, that we will not repeat here what our readers may already know. The question as to whether it is situated between the mucous membrane and the epithelial layer, its chemical composition and microscopical appearances have but little value to the practical physician, as there are other more certain and ready means of diagnosis. That the false membrane has an erosive or irritating property, is exemplified by the following case:

*Case.*—Girl aged 7 years, was attacked with a mild form of diphtheria. The local symptoms yielded readily to treatment, so that on the third day there remained but a small patch of the false membrane about the size of a half dime on the left tonsil. Wishing to watch the process of spontaneous detachment we left it undisturbed. On the morrow it had increased in extent, and on the third day it covered nearly the whole tonsil, and the cervical glands had notably increased in volume. Deeming it imprudent to let it remain, it was carefully detached with the forceps and an ulcer was discovered on the tonsil corresponding to that portion of the false membrane which had remained the longest. The local application of the sesquichloride of iron, and this remedy with chlorate of potash soon produced cure. Although the tumefaction of the cervical glands existed in every instance, yet we think that their rapid increase in volume being coincident with the existence of an ulcer in this case, will illustrate that dependency between this enlargement and the absorption of some irritating or poisonous secretion. This relation is analogous to what is seen in other diseases, as between a chancre and bubo, and between enlarged mesenteric glands and ulcerated peyer's glands. The existence of the ulcer we are inclined to believe depended upon the long continuance of the false membrane upon the mucous surface, as the same lesion was noticed in another case where the exudation had evidently existed some days before we saw the case. May not the infrequency of ulceration depend in *some measure* upon the local treatment now generally adopted and so necessary in the management of the local symptoms?

*Gangrene.*—This occurred but in one instance, and this was the first case and first victim of the epidemic. The following is the case as related to us by Dr. Clark:

*Case.*—A boy 10 years of age, had been sick for six or seven days before I was called. He was already in an alarming con dition, there was great prostration, his face œdematous and of a cachectic leaden hue, pulse quick and feeble, a complete inability to swallow, a quickened but not efficient respiration, breath fetid, and sides of the neck swollen, and a fetid diarrhœa. On examination of the throat there was discovered a loss of substance of both tonsils. They were excavated, presenting a bloody and greenish appearance, and numerous shreds of filaments of the fauces presented a dirty ash color, and the odor was not to be mistaken, so characteristic was it of gangrene. From the general condition of the patient, the extensive local ravages of the disease, and the loss of the power of deglutition, I at once pronounced the case fatal. My best directed treatment had no influence on the gangrenous destruction of the parts, and on the fourth day the patient died with almost a total destruction of both tonsils, the uvula and part of the soft palate; the whole of the fauces appearing as a continuous gangrenous ulcer. I was in doubt as to the nature of this disease, so rapid and malignant was it in its progress, until the day before his death when another of the family was taken sick, bearing the unmistakable marks of diphtheria. The disease ran rapidly through the family, four children, mother and domestic, sparing the father. We will refer to this case hereafter.

*Lymphatic glands of the neck.*—A tumefaction of these glands always accompanied the inflammation of the throat. This was a constant symptom, and was most manifest on the side where the throat was mostly inflamed or the false membrane most extensive. In a few instances this tumefaction of the glands existed before the exudation was detected, but this latter did not delay to make its appearance. But in these cases, it is possible that it may have existed, and became detached by coughing or in the effort of deglutition prior to examination. This glandular swelling would remain for a variable length of time after the disappearance of the false membrane; in no instance did suppuration supervene, a fact noticed by some observers.

*Fever.*—The febrile symptoms varied greatly in different cases. These seemed to be governed more by constitutional peculiarities rather than by the severity of the disease; ordinarily, however, they were but slight. In some cases during the whole course of the disease, the febrile movement would be scarcely appreciable, in others it would be more or less intense from the beginning.

*The Tongue* was almost invariable in its appearance. It presented a dirty white or cream colored coating, through which the enlarged and red papilla projected, giving the organ a very peculiar appearance.

*A want of Appetite* was one of the most constant and troublesome symptoms, often amounting to a *great disgust* for food. In many instances this would continue not only during the activity of the disease, but also during convalescence, baffling every attempt on our part in the administration of remedies, and the ingenuity of parents in the preparation of dishes to excite it.

*Voice.*—In many cases there was a marked alteration in the voice, it having a decided "nasal twang," nothing in common however, with the hoarse and whispering voice of croup. This did not depend upon obstruction of the posterior nares by any tumefaction of the parts, but it could be referred to a paralysis of the soft palate.

*Cough.*—This when present, was neither violent nor paroxysmal, but short and expulsive, as if to free the throat from some irritating substance.

*Albumine in the Urine.*—Albumine was present in the urine of some of the cases examined; quite abundant in one of the fatal cases. Knowing the importance of this symptom in other diseases, we regret that we did not carry our investigations so far that they might be of some practical value. The fact, however, that it was not constant in all of the *few* cases examined, is sufficient to demand for this symptom a more careful appreciation in future observations.

*Symptoms referable to the cutaneous surface.*—The skin presented but one important symptom, and this was the false membranous exudation. The following case will illustrate the importance of this symptom.

*Case.*—In a family of five children two were confined to bed with diphtheria. At one of our visits we noticed a third, a boy aged 8 years, with a pale countenance, a coated tongue. He complained of a loss of appetite, head-ache, and had vomited twice during the twenty-four hours. On examination we found the fauces slightly inflamed; absence of false membrane, and no tumefaction of the cervical glands. These symptoms were looked upon as prodromata, and we told the family that he was suffering from the epidemic poison. On inquiry as to whether he had any sores on his body, we learned that he had a few days previous injured his foot, and on examination we found a sore covered with a diphtheritic exudation, and the surrounding integuments had the appearance of an erysipelatous inflammation. There was now no doubt as to the cause of his indisposition. Two days after, the sore had nearly doubled in size, and on the third day the false membrane made its appearance in the throat, thus making the case pharyngo-cutaneous diphtheria, according to the universal and erroneous manner of naming this disease from one or more of its local manifestations.

This case is but a type of many met with, but in none was the exudation confined to the cutaneous surface, and here it only appeared in those parts already deprived of the epidermis, whether by disease, accident, or remedies in one case on a blistered surface.

This case conclusively illustrates that the symptom or sign which has given name to this disease, is but a local evidence of a morbid poison affecting the whole system, having a predilection however for its manifestation in the throat, and requiring certain conditions of the skin, an abrasion, ulcer, etc., for its appearance on this surface.

Besides the above, there are constitutional symptoms, which, when mentioned by authors, have been passed over with too slight a notice. In these lie the *genie* of the disease, they alone being sufficient to stamp its individuality. Too much importance has been given to local manifestations; had the same attention been given to that general condition of the system tending to or producing their development, much of the confusion as to its specific character and pathology would have been avoided. We refer to a more or less intense anæmia, a depression of the vital powers, and to local or general paralysis. These conditions, when spoken, have been described as sequences, but that they may appear during the activity of the disease, or even constitute the only evidence of the action of the diphtheritic poison, we have indubitable testimony.

*Anœmia.*—There was a constant tendency to an anæmic condition; in some cases it was the earliest symptom noticed. It generally made its appearance early in the disease, and in some cases it would not arrive at its maximum until long after the disappearance of the false membrane. In several instances, in families where diphtheria was prevailing, cases of sudden anæmia occurred not referable to any appreciable cause, nor accompanied by the pathognomonic sign of diphtheria, but the same condition being common in well marked cases of diphtheria, we could not but conclude that it depended upon the influence of the prevailing morbid poison. The characteristics of this state are too well known that it should need a description from us, its only peculiarities in diptheria being its occasional sudden appearance, its rapid progress, and absence of common causes.

*Nervous depression.*—This state, like the preceding, varied as to the stage of the disease in which it made its appearance, usually it was an early symptom. It was also a common symptom, but differed in its degree in different cases. This prostration of the constitutional forces bore a strong resemblance to that observed in typhoid fever; the patients, though silent, inattentive, restless, sleepless, feeble, and fatigued, did not present that stupor or delirium of typhoid. We will again speak of this state in our remarks.

*Paralysis.*—Paralysis of the soft palate was observed in many cases, the patients having a decided "nasal twang" of the voice, and more or less difficulty of pronounciation; in one or two cases this was accompanied with paralysis of the muscles of deglutition, as shown by the regurgitation of food by the nostrils in attempts to swallow.

*Hemiplegia.*—This occurred but once.

*Case.*—A boy aged 5 years, in excellent health, was attacked with diphtheria during his brother's sickness with the same. The disease passed through a mild course. The false membrane ceased to appear about the fifth day, and convalescence seemed to have been established. His appetite improved, his former playfulness returned, and he engaged in his ordinary nursery sports. In about a week we were again summoned, and found him with a pale and puffy face, quick pulse, a distressing sensation of fatigue, loss of appetite; no soreness of the throat, no pain in deglutition, no appearance of false membrane. We attributed his present state to imprudence on part of the parents, in permitting him to play in the yard improperly clad—head uncovered and face bare—exposed to a damp atmosphere. Two days after, we found him in a profound adynamic state, the anæmia intense, face pale, of a leaden hue, and somewhat œdematous; the anæmic bruit audible over the cardiac region and in the carotids; no head-ache; the cerebral functions undisturbed. *There was a decided nasal intonation of his voice, food regurgitated by the nostrils, and on taking him out of bed we discovered that he had lost the use of his lower limbs.* Albumine quite abundant in his urine. These symptoms became more and more marked, notwithstanding our best directed energies; and he gradually sunk, and died on the sixth day of our visits, from extreme prostration.

This case, although imperfectly given, is instructive in more than one point. Here we find combined in the same patient two of the most formidable sequela of this disease, the one common and the other exceptional; and the time of their appearance shows that the cessation of medical attention and treatment should not be determined by the disappearance of the false membrane, nor by an apparently well established convalescence. We will give another case illustrative of this fact when we speak of the treatment. It also shows the error of giving too much stress to the local manifestation to the neglect of constitutional phenomena, which may constitute the only evidences of the disease. This rapid and fatal prostration may occur before special lesions are manifested.

*Duration.*—When the disease was without complications, it ran through its stages in a few days. However, there was nothing constant in this. The false membrane disappeared in some cases in two days; in others from ten to fourteen days. In cases complicated with anæmia, the convalescence was slow and long; a few still under treatment, three months from date of sickness, for this condition.

*Relapse. Case.*—A young lady, aged 19 years, was attacked during the early period of the epidemic. The disease was well characterized, pursued a mild course, and disappeared in a few days. During her sickness she was suffering from disordered catamenia, and she presented the appearance of one laboring under incipient chlorosis. This suppressed menstruation added to a mental depression caused by an *annoying disappointment*, gave such an impulse to the tendency of her diphtheritic attack to anæmia, as to demand special attention. She was put on a tonic and chalybeate course. Four weeks after, her angina ceased, and at the time when the epidemic so suddenly increased in violence, she complained of head-ache, an increased loss of appetite, and soreness of the throat, which soon put on the distinguishing marks of diphtheria. The relapse was more severe and longer continued than the first attack. Her convalescence was incomplete, she having suffered long from paralysis of the soft palate; the only inconvenience, however, being an alteration in her voice and she is still, four months from first attack, under treatment for decided anæmia.

Whether this case should be considered as an instance of second occurrence, or whether the two attacks were but the one and same disease, the morbid poison having ceased to operate for an interval, we will not attempt to decide positively. After her first attack, or during the interval of quiet, which was also the interval of repose of the general epidemic influence, her throat resumed its healthy look, her tongue cleared, and the cervical glands resumed their normal condition; there were no traces of diphtheria, save her anæmic state, which could be ascribed to other causes. Added to these facts, the length of time between the attacks, and the sudden and violent manner of the second outbreak of the epidemic, we are inclined to regard it an instance of second occurrence, her health establishing a predisposition which yielded to the causes which renewed the force of the prevailing influence. To regard it as a case of second occurrence would not militate against the specific character of the disease. This is but what occurs in other diseases where specific character is unquestionable, as in measels, scarlet fever, small pox, typhoid fever, etc. If this were or were not a fact, it would have no weight in establishing the peculiarities of diphtheria; "these must be facts determined by experience, and not inferences determined by reasoning."

*Treatment.*—We need not dwell long on the treatment, as we claim no originality in the method pursued. Of all the internal remedies that have been recommended, we placed our chief reliance on the tincture of sesquichloride of iron, hydrochloric acid, and chlorate of potassa. These were either given together in some convenient vehicle, or in alternate doses. Occasionally, but rarely, the tincture of iron could not be borne for many successive days; in these we gave as a substitute, sulphate of quinine and citrate of iron. The doses of these remedies were full, and regulated according to the age of the patient. Emetics were given at the onset in some cases, but as they did not seem to have any effect on the severity or duration of the disease, they were discontinued, except in those cases where their mechanical aid in detaching the membrane would be of service. When cathartics were called for, the mildest were selected; in those cases where enemata would answer, they were always preferred.

A generous died was allowed; beef tea, mutton broth, rich chicken soup, etc., together with a more or less free use of wine, brandy, or any other stimulant at hand. We urged, and in some cases forced patients to take nourishment. notwithstanding the loss of appetite, amounting in some cases to a disgust for food, as these symptoms were no index as to the state of the digestive powers.

*Local Treatment.*—This consisted in the removal of the false membrane *with the forceps*, which, except in the youngest patients, could be easily done by devising means of keeping the mouth open and tongue down, and the subsequent application of a solution of the nitrate of silver, twenty to forty grains to the ounce, the strength varying according to the severity of local symptoms. The tincture of the sesquichloride of iron, or hydrochloric acid, were substituted for the nitrate where there was an impossibility to remove the membrane, believing that they assisted more in detaching the membrane than the nitrate; they were also used whenever there was any fetor or suspicious discoloration of the membrane. The frequency of these applications was determined by the rapidity in which the membrane was reproduced. Gargles of tannin, alum, chloride of soda, and chlorate of potass were used; the two former seemed to be useless, the latter acted undoubtedly as a solvent of the membrane; this we have frequently proved by placing portions of the membrane in a strong solution of the chlorate.

Both general and local treatment should be continued for a few days after the disappearance of the false membrane, as the following case will illustrate.

*Case.*—A boy, five years of age, had suffered from the ordinary symptoms, and was treated in the usual manner. On the fifth day all traces of the false membrane had disappeared. With general directions as to his management, and with strict injunctions that his throat should be examined daily, we left him as convalescent. Three days after we were again summoned, and found the exudation covering both tonsils and part of the soft palate. Patient slowly recovered.

Thus have we attempted to describe the progress and phenomena of this epidemic as faithfully as possible, and as minutely as is necessary. We could here conclude our article, leaving the reader to draw his own conclusions, but there are certain facts bearing upon some disputed points as to the nature of this disease, which we do not wish to pass in silence.

*Remarks.*—Every few years the attention of the medical world is arrested by the discovery of a new disease, and our journals are gorged by writers who wish to print, to do honor to the discoverer, and to swell the importance of the strange disease.

Ever since M. Bretonneau, of Tours, published his observations on the disease, the discussions as to its nature have been continued, so that there is no disease in modern times whose literature is so rich, and we may add so confused. That the disease has not been fully understood, we may judge from the multiplicity of names it has received ; perhaps there is no malady whose nomenclature is so extended and so vague. From this confusion in nomenclature and descriptions arises the opinion of some that diphtheria is of recent origin.

*Is it a new disease?* We do not intend to give more of the history of diphtheria than is necessary to answer this question. We are loath to believe in the " sudden development of strange types of disease " of new organic poisons and new contagions. The notion of a new disease springing up among the human race militates against that law of sameness observable in their economy. A first recognition and description of a malady particularly constitute its newness, but this is no proof that it has not existed in all ages. Diseases are often overlooked or confounded with others ; descriptions drawn from hasty inferences ; theories imperfectly conceived, and facts obscurely ascertained, pass through lengthened periods of our art, and it is the separating the dross from the metal that constitutes much of our progress. Whooping cough was not described as a separate disorder prior to Dr. Thos. Willis, and we learn from Willan that Sydenham was the first to mention scarlet fever, yet no one doubts but what these diseases existed before these observers ; we only think it strange that they should have passed so long unnoticed. It is stranger still that diphtheria, terrible and destructive as it has been in some countries, should not have been recognized by the profession at large as a disease *suigeneris*, long ere Bretonneau pronounced it such.

We find in ancient and modern writers descriptions of it, as graphic and clear as that given by this noted observer. Indeed he does not claim the title of discoverer, for he traces descriptions of diphtheritic inflammation from the time of Hippocrates down to his own age. The following passages from Areteus are sufficient to show his acquaintance with this disease. "Ulcera, in tonsillis fiunt, aligna mitia, aligna pestifera, necantia. Pestifera autem sunt lata, cara, *quodum concreto humore albo, livido, aut nigro sordentia.* * * * *At si in pectus per arteriam id malum invadit, illo eodem die strangulat.* Pueri usqne ad pubertatem maxime hoc morbo tentantur."—(*Medico*

*Chir. Review*, new series, vol. 5, p. 427.) One cannot fail to recognize in this quotation a good description of the pathological peculiarities of diphtheria, and of the manner in which it frequently proves fatal. At a date nearer our own times, writers on this disease become more frequent; we will simply mention the names of Morton, Cullen, Cotton, and Huxham; these, however, confounded it with measles, or scarlet fever. Fothergill, in 1748, "was the first to describe as a *new and separate disorder*, that perilous form of the complaint which Cullen designates cynanche maligna, and it was long called the Fothergill sore throat."—(Watson.) About the same epoch other writers described this disease as a *primitive* affection. Among them Starr and Ghisi were the most prominent. But this step toward progress was soon lost, for we learn from the same author just quoted, "that the identity of this affection with genuine scarlet fever has been slowly established by subsequent observers!" But our countryman, Dr. Samuel Bard, who published his observations in 1771, did more than any preceding author in delineating this malady. He was the first to recognize the nature of the local lesions, and so full and just are his opinions, that subsequent authors have added but little to his description. He pointed out the connection between the angina and croup, and the manner in which the air passages were invaded by the false membrane. He gives observations of the angina alone, of croup alone, and of these diseases complicating each other, considering these, however, as identical. He gives the differential characteristics between this disease and pultaceous pharyngitis, showing that the appearance of the throat was not the result of gangrene, and regarding the false membranous patches as the result of a concretion. Bard was soon forgotten, and everything touching this disease continued vague and confused, until the distinguished Bretonneau published his researches. His delineation was so clear and minute that the disease became easily recognizable, and epidemics were observed almost simultaneously in different parts of the world. If we possessed no evidence of the antiquity of the disease, its history since Bretonneau's time would indicate that it has existed in all ages; for it would seem absurd to believe a disease of recent origin, should, so soon after his description, appear in different countries where climate, condition of the inhabitants, etc., were so diverse.

In the *London Lancet*, (article Diphtheria, April 1859, page 203,) it is stated, "one practical fact is indeed well established, that it is a disease until lately unknown to the practitioners of this country (England), and not formerly described by any of our older writers. The affections so well known and so vividly described by Fothergill, Huxham and other physicians were undoubtedly the scarlatinal angina and the angina gangrenosa, or malignant cynanche, but dyphtheria is separated from them by a line of demarcation, at least as strongly distinctive as that which divides diarrhœa, dysentery and cholera." This is the tone of all the present English writers. Is the disease mentioned by any of their older authors? Dr. Starr, described an epidemic of angina maligna which raged in Cornwall in the year 1748 under the name of *Morbus Strangulatorius.* Dr. Fothergill, whose treatise on the malignant sore throat appeared in 1748 says, "that great difficulty of breathing" took place in all previously to the fatal termination. In Huxham's account of the epidemic of 1752—3 the same termination is distinctly noticed. In speaking of the expectoration of what he termed the *sloughs*, he says that a piece of the *internal membrane of the wind pipe* was discharged, meaning (the false membrane of course.) Dr. Withering says that "the affection of the fauces in some cases spread itself down the wind pipe of the lungs, as was evident from the cough, the strait breathing and other peripneumonic symptoms." In Dr. Cullen's account of the cynanche maligna, it is stated that from dissections it appears that in C. malig. the larynx and trachea are often affected in the same manner as in the C. trachealis, and it is probable that in consequence of the affection, the C. maligna often proves fatal by such a sudden suffocation as happens in perfect cynanche trachealis." (*Laennec*, American Edition, note. p. 126.) As the symptoms described by the above authors are common in fatal terminations of diphtheria, and very uncommon in scarlatinal angina and angina gangrenosa, we need not stop to show they were acquainted with this disease, though not favorably described by them. In our own country since Bara's time, the disease has been noticed and described under the names of putrid sore throat, the sore throat epidemic, malignant sore throat, scarlatina anginosa sore throat.

*The confusion arising from the fact that fatal cases have been given as types of the disease.*—From the history of epidemics as observed in France, England and elsewhere, the present would be considered as of a mild character. We attribute much of its mildness to early treatment. During the epidemic, parents would daily examine the throats of their children, and on the slightest indisposition we were summoned, so that with some exceptions we met with the disease in its forming stage. This variation in severity is observed in all epidemic diseases, and we fear that accounts of epidemics of diphtheria, as have prevailed in Europe, have been of the most malignant form. Bretonneau recognized a mild form, to which he gave the name of "*angine couenneuse commune*," and endeavors to show that it is a bastard affection, but from the description he gives of it, we are unable to distinguish it from true diphtheria. He bases the difference in these forms upon the progress and result of the disease, if it progresses without any complication and terminates favorably, it is the "*couenneuse commune*," but if its march is rapid sequela, or death ensue, then it constitutes his *diphtherite*. We do not admit however, of this distinction, it is giving to *diphtheria* a constancy in its type which is at variance with those laws which govern epidemics generally. This class of diseases are limited to no climate, and the phenomena they present may vary with the localities in which they appear; consequently no observer, however attentive he may be, can acquire a full and accurate knowledge of them from personal observation alone. The disease as Bretonneau observed it, was no doubt generally of a severe type, as in the whole of them who were examined, post-mortem, the false membrane which constitutes croup was found; from this, and from the fact that he considers this mild form a spurious affection we can justly draw the inference that he considered croup as almost an essential symptom, whereas it is but the most constant fatal termination. We are slow to suspect the accuracy of so distinguished an observer, but our motive is honest when we say, that from the constancy in which we meet with croupal symptoms in his reported cases, he may have overlooked that which would militate against his theory of the identity of croup and diphtheria. From this error arises the common belief that diphtheria is in the majority of cases almost necessarily a fatal disease; those cases mild in form, free from complications, and of favorable termination being overlooked, or regarded as some other form of angina. It is then, from accounts of several cases, and reports of epidemics of virulent fatality, that diphtheria has received the name of a terrible and formidable disease, and so universal is this opinion that reports of mild epidemics are received with suspicion as regards their honesty and accuracy.

There is another class of observers, who having never witnessed the severer forms, adopt an opposite extreme, and look upon diphtheria as only a variety of ordinary sore throat, deriving its fatality from such local circumstances as would intensify any prevailing disease. It has been also called a *malignant* disease, but except where it occurs as an *intercurrent*, or when it terminates in gangrene, we cannot see the justness of this appellation; in the former its malignancy has some connection with the reigning epidemic, in the latter it depends upon the individual constitution or the intensity of the local affection.

*The importance of the false membrane as a symptom.*—Whatever may be the diversity of opinion as regards the nature of diphtheria, by far the greater majority of writers agree that the membraniform exudation is an essential constituent of the disease. Bretonneau remarks that "this redness of the mucous membrane, without thickening of its tissue, so superficial, and yet accompanied by a concrete exudation so abundant, appears to him so remarkable as to deserve the name and character of a specific inflammation, *sui generis.*" He therefore, designates this phlegmasia by the term *diphtherite*, from the Greek, pellis, exuvium vestis coieacea. Whatever other appellations it may have subsequently received, they all refer to the throat symptoms, the presence of which marking the active stage of the disease, and their disappearance on approaching convalescence. But still a greater importance than being a constant symptom is given to the throat affection, "*it constitutes the principal malady*, and the gravity of the affection depends but upon the extension of the false membrane into the air passages." (*Guersant Dict. de Medicine.*) Trousseau, Barthez, Rillet and Valleix, have expressed the same idea in almost the same language. These authors have cited cases where death has taken place without croupal symptoms, but in these "there existed a cutaneous diphtheritis more or less extended, which seemed to announce a general infection sufficient to explain the gravity of the affection."—(*Valleix.*) The fact that diphtheritic inflammation of the skin can exist alone, has led some authors to make this a distinct variety, and when it co-exists with the throat affection, it constitutes a serious complication. The severity of the disease has been determined by the extent of the false membrane, and the rapidity of its reproduction.

Not only has the false membrane received this exalted importance in diagnosis and prognosis, but it is the principal, and with some the only symptom necessary to combat. Thus being more apparent and more constant than other symptoms not less important, it has received a more prominent place in descriptions.

*Does the appearance of false membrane determine the duration of the prodromic stage, or is it at all essential to the full activity of the disease?*—If any morbid condition, prior to the production of the false membrane is to be considered simply as precursory evidences, we, perhaps, in describing the present epidemic, should have mentioned under the head of prodromata, a prostration of the vital powers, a more or less intense anæmia. When these did precede the exudation, the cases were of a severe form, and according to other observers, they are in themselves sufficient to hasten a fatal termination.

The symptoms that we have given as prodromata, would more properly constitute the first stage of the developed disease. The system is already under the active influence of the diphtheritic poison, and they do not necessarily indicate that a false membranous exudation is to follow. It is erroneous to consider the exudation always an essential element, marking the different stages of the disease; it is but an epiphenomenon, a local manifestation of a local poison affecting the whole system—irregular in its appearance as to the stage of the disease, it may be the first evidence, *or it may not exist at all.* The first stage may be of such severity as to confine the patient to bed, and demand active interference on the part of the practitioner, and yet recovery may take place without the appearance of the exudation. We need go no farther than our own observation to prove this.

*Case.*—A stout healthy boy, 14 years of age, was, at the time when three in the same house were suffering from well-marked diphtheria, seized with the usual premonitory symptoms—chill head-ache, vomiting, coated tongue, redness of the fauces—which were soon followed by a general feebleness, pale countenance, weak, though not rapid pulse, and a feeling of malaise to such a degree that he sought his bed or lounge for comfort. This state continued for about a week, when he gradually returned to his former health. Careful and repeated examinations of throat and skin revealed no false membrane. We could give several similar instances, and the phenomena being identical with those present in cases where the false membrane existed, we were compelled to ascribe them to the same cause, the diphtheritic poison. (These cases were *not* included in the 133 reported.) In these cases general and local treatment was adopted; this may have had much to do in preventing the formation of the false membrane, if so, it shows the necessity of giving full importance to the first manifestations of the disease. In sporadic cases the presence of the membrane is necessary to a positive diagnosis, but during the prevalence of an epidemic, uniformity in the other various phenomena is sufficient to guide the practitioner.

In an excellent and valuable report on diphtheria in the March, April and May numbers of the *London Lancet*, we find that in different epidemics "death occurred in many cases in the first stage through sudden and extreme adynamia, *before the exudation had fully formed.*" This is not an unfrequent occurrence in the experience of others, as is seen from reported cases in our journals. This statement would lead the reader to suppose that if death took place so early, there was *not time*

*for the exudation to form*, but it shows that it played no part in the fatality of the disease.

*The severity of the local phenomena bears no relation to the severity of the general affection, nor to the character of the convalescence.*

There are cases in which the "local manifestation" is from the first overshadowed in importance by the constitutional symptoms, and *vice versa.* This was particularly true in the present epidemic. In some cases where the local manifestation was far from being intense, yielding readily to treatment, the constitutional symptoms were severe, and convalescence long and embarrassing. In two of the fatal cases the local symptom was early and easily checked, and was so mild during its continuance as to give but little annoyance either to patient or physician. These cases proved fatal by a general sinking of the powers of life. In other cases this state of affairs was reversed, severe local symptoms with no corresponding constitutional disturbance, and rapid convalescence.

However mild or severe, then, may be the local affection, "the constitutional symptoms form a very large and important part of the morbid manifestations to combat. Moreover, these constitutional phenomena have this peculiarity, that not only do they manifest their presence at the outset of the disease—often, indeed, with such severity as to destroy life before the local or special epiphenomena have had time to develop themselves—but they also tend to show themselves at a very advanced period when the local disorder has passed away, and when, in many respects the patient might otherwise be considered to have recovered from his malady, and to have reached that period when serious results were no longer to be dreaded."—(*London Lancet*, Sept., 1859 p. 214.)

*Diseases analagous to diphtheria.*—All those diseases which present prominent throat symptoms, with a pseudo-membranous exudation, have not only been considered similar, but identical. But to prove the identity of disease it is necessary there should be more than one mark of resemblance or familiarity, there must be some other analogy in their general appearance and effects. The general name of a disease seldom gives us a true appreciation of its nature. A disease may differ from itself, so to speak, and when it takes its name from a prominent symptom, yet common to other diseases, divisions and distinctions are made which soon leads to a confusion of diseases between which there is not the least analogy. The diseases with which diphtheria is considered by some to be identical, are, croup, pultaceous pharyngitis, and angina gangrenosa; in considering these we will include all we wish to say on the subject of diagnosis. The ordinary forms of tonsillitis and pharyngitis present local and general symptoms so diverse from the disease under consideration, that they demand no notice from us, and laryngismus stridulus, trachitis, and laryngitis, can only be confounded with croup, which we consider essentially distinct from diphtheria.

*Croup.*—Since Bretonneau's work on diphtherite, the identity of angina membranacea with croup, has been considered a well established pathological fact. Authors have received his conclusions as indisputable, and they, substituting his observations for their own, freely borrow from his pages the details for their descriptions. Croup is rarely described as an isolated affection, it being considered a phase or period of pharyngial diphtheritis, the single fact of the presence of the false membrane in both, establishing their identity.

"M. Bretonneau has demonstrated that epidemic angina maligna is a true pellicular inflammation like that of croup. He has also proved that these two morbid alterations are identical in their pathological anatomy, differing but in the seat they occupy."—(*Geursent Dict. de Medicine*, t. 9, p. 336.) "Is it necessary to say that the skilful physician of Tours, has proved in the most positive manner the identity of the different pseudo-membranous inflammations of the mucous and cutaneous surfaces, designated by the names of angina gangreno sa, croup, etc."—(Barthez and Rillet.) This very identity, contended for by Bretonneau, was fully and distinctly stated before him by Dr. Bard in 1771, and by Dr. Jas. Johnstone, 1779. The latter says, "There is but one other species of angina from which this disease (ang. maligna) requires any distinction, and that is croup. A small degree of attention to the several divisions of that distemper, which have been made by the best writers, will show that *in respect to many of the cases there can be no distinction, for in reality there is no difference.*"—(Laennec on the chest, p. 126, note by Trans.)

Thus we see that not only French, but American and English authors have endeavored to identify diseases which are opposite; each of these observers, however, arrived at their conclusions from observations made during epidemics of angina maligna.

Bretonneau, whose researches into the phenomena of ang. malig. have been more minute than those of any other pathologist, proves this identity from the result of fifty-four post-mortem examinations. In these he twice found the false membrane confined solely to the air passages, while in all the other instances, the disease invariably commenced in the pharynx, and presented symptoms of ang. gangrenosa. "If we consider,"

says a reviewer, "that in the whole of those who were examined, *post.mortem*, the false membrane which constitutes croup was found in the larynx, and if we consider that the same fact under the same forms and attended by the same circumstances, were constantly observed by Dr. Guersant, and also by Dr. Velpeau, we shall be strongly urged to grant that angina ma ligna and croup, are the same disease affecting different portions of the same mucous membrane." This is only a statement of the invariable presence of a disease, the nature of which is in question, and the conclusion arrived at is only a proof that the croupy affection is the common fatal termination of the anginose affection, and it is from fatal cases alone that Bretonneau endeavors to establish this identity. He rejects this method of reasoning when he wishes to prove the specific character of diphtheria. Thus, when croup complicates a febrile affection, as measles, scarlet fever, etc., it is no longer *croup*, this being an expression of that morbid state to which he has given the name of diphtherite, and having a specific character; whereas secondary pseudo-membranous laryngitis is only specific in so far as it borrows this character from the disease it complicates. From the manner in which he proves the identity of ang. malig. and croup, we cannot see how he can make this distinction, for both present the same resemblances in the nature of the false membranous deposit and in the coincidence of its appearance upon the mucous membrane of the pharynx and larynx. This difficulty, perhaps, presented itself to his mind, when he says, "that the false membrane alone is not sufficient to characterize croup." Had he recognized this fact when endeavoring to prove the identity of diseases so opposite, he would have searched for other analogies than this one gross resemblance. Although he acknowledges the existence of croup commencing in the larynx or trachea, he insinuates that these causes may not be true diphtheritic inflammation. "He asks as if he feared he were pushing his opinion of the identity of these diseases too far, whether it does not happen that concretions other than those of a diphtheritic nature, are not developed in the larynx."—(*Valleix.*)

Other authors, with him, assert that the membranous angina is almost essential to croup, the angina constituting the first period. "If croup, instead of commencing in the pharynx, commences in the larynx, which is rare, or in the trachea, which is still less frequent, the first period of the disease is wanting."—(*Guersent.*) But how explain the fact that epidemics of croup do occur where the presence of the membranous pharyngitis is the exception and not the rule? On the other hand, how explain the fact that epidemics of diphtheria occur and none of the cases present croupal symptoms? Why is it only in the epidemic form that croup begins with the pharyngial affection, whilst in sperodic cases it debutes by the larynx? What is there in the diphtheritic poison, that it should change its seat of manifestation in different epidemics? That it should at one time produce an epidemic of the 'first period,' and at another, of the 'second period' of the disease?

There are other more positive differences between these diseases.

*They differ in their nature.*—Croup is an acute inflammation of the larynx or trachea, or both, characterized by an exudation of false membrane. It is *purely* an inflammatory disease of a highly acute character—the symptomatic fever severe, pulse accelerated, skin hot, etc.—and as such it is described by those who do not deny its identity with ang. malig.

Angina maligna is essentially an asthenic disease, the fever of a typhoid character. "It is accompanied from the first by a depression which seems to be in many respects peculiar, and to approach nearer to the pure asthenia than anything witnessed in other cases of acute disease. "The constitutional symptoms present nothing in common with those of croup. This fact did not escape the notice of those who contend for the identity of these diseases, and the summary and easy manner in which they explain away this *capital* difference between them is anything but satisfactory". Croup, *even when most partial*, is almost always accompanied by great constitutional disturbance, symptomatic fever acute and very severe, etc. In some cases, particularly such as occur in hospitals, the state of the system is very different, there being evident marks of a septic change in the fluids of the body; the pulse is but little accelerated, the skin harsh and dry, the debility extreme, and the breath fetid even where no gangrenous specks exist." This variety is denominated *asthenic* by Guersent and Bretonneau.—(Laennec, disease of the chest, p. 128.) There can be no doubt that ang. malig. is referred to in the above, as he, in the following sentence, speaks of the character of the false membrane, "especially that lining the throat." "When croup is connected with inflammation of the tonsils, soft palate and fauces, and the deposit of false membrane upon them, the disease is of an *asthenic* character."—(West, dis. of children, p. 224.)

If there is no essential difference between these diseases, if they are of the same nature, and depend upon the same cause, we cannot comprehend why croup should be *sthenic*, when the false membrane begins in, or is confined to the larynx and trachea, and *asthenic* when it is first formed in the pharynx. It seems absurd to attribute to this slight variation of one to two inches in the seat of the local manifestation, and this upon the same continuous membrane, a power of producing opposite conditions of the system.

Again, it is said by Bretonneau, Guersent and others, that *asthenic* croup is contagious, but that this is true in regard to croup in general, is a question still undetermined. This admission is unfortunate to their theory of the identity of the two diseases. We could have produced instances, when giving the history of the present epidemic, proving that diphtheritic pharyngitis is contagious, but we did not think our evidence could strengthen a fact already well established. We cannot but exclaim with Dr. Cheyne, "Asthenic Croup." Croup with an unaccelerated pulse, fetid breath, and propagated by contagion!

*The difference in their symptoms.* Besides the difference in constitutional symptoms already spoken off, there are differences in one or two local symptoms, which we will but briefly hint at.

*Swelling of the Lymphatic Glands of the Neck.*—Britonneau makes the absence of this symptom a distinguishing characteristic between *genuine* croup and laryngismus stridulus. It is indeed a constant attendant upon ang. malig., but that it is absent in tracheal croup, we need but give the following quotation. "The swelling of the cervical lymphatic glands, so common in diphtheritic pharyngitis, is wanting when croup commences iu the larynx. In the epidemic observed by Vauthier, it was noticed but once in 37 cases. At Geneva it is rarely ever observed, and this has been the experience of one of us in the sporadic cases seen in Paris."—*Barthez and Rillet.*

*The circumstances attending the development of cutaneous diphtheritis are in themselves sufficient to defferentiate diphtheria from all other disease.*—It is in those cases where the pharyngitis exists with all its diphtheritic characters that the exudation is most liable to appear on the skin. Any cause that deprives this surface of its epidermis becomes the occasion of the development of the false membrane. In genuine croup this is never observed. Do blistered surfaces and leach bites, in cases of croup, put on this morbid action? Experience in these remedies answers this question in the negative. These remedial measures are recommended in croup by those who claim the identity of these diseases, but they are strongly denounced by the same authors in diphtheritic pharyngitis, as they produce a "dangerous complication"—a cutaneous diphtheritis.

*They differ in their treatment.*—The treatment in the one is decidedly antiphlogistic, in the other it is essentially tonic and stimulant.

"I have never met with an exception to the rule which prescribes the free abstraction of blood in every case of severe idiopathic croup, and you must bleed largely and give tartar emetic freely, for these are the two measures on which your main reliance must be placed. Much good can be done by blisters, &c."—*West, page* 224. Yet this author cannot see that there is any ground for supposing there to be an essential difference between croup and the affection described under the name of diphtheritis by Bretonneau. He adds, when croup begins with a false membranous deposit in the fauces, the disease is of an asthenic character, *and a corresponding modification must be made in the treatment.*" What these modifications are we need not repeat here, suffice it to say that this very 'modification' proves the diseases different in their natures.

*The difference in the results of the operation of tracheotomy* in these diseases is another distinguishing mark. We copy the following from notes taken by ourselves, a few years ago, in the wards of the "Hospital des Enfans Malads," Paris; "The presence of false membrane in the throat, nares, or on the skin, strongly counter-indicates the operation of tracheotomy." If the false membrane in the trachea is the only cause of death, why would not its extraction be followed by the same result in either case? We believe, without having any positive proof for the assertion, that in croup there is not that tendency to rapid reproduction of the false membrane as is seen in diphtheritic pharyngitis. We know that if the false membrane of croup is thrown off, the tendency is to recovery; but in the pharyngitis, notwithstanding frequent caustic applications, the membrane rapidly re-forms, and this continuing in cases for many successive days.

*They differ in their convalescence.* In *croup*, when the mechanical obstruction is removed, and the case terminates favorably, the return to health is rapid; while the convalesence of the other disease is slow and uncertain. Long after the disappearance of the false membrane there may be developed morbid manifestations, in themselves sufficient to cause death.

*Scarlatina.*—The confusion between diphtheria and scarlatina is still so common, that we consider it worthy a moment's consideration. From a rude resemblance in the throat symptoms, from the not unfrequent coincidence of scarlatina with diphtheria, and from the fact that it is occasionally complicated with a pseudo membranous laryngitis, arises the error that they are closely allied. However, there are some cases of scarlatinal angina which so closely put on the aspect of diph theria, that it is impossible to distinguish them. Guersent and others have met with such exceptional cases.

Pultaceous pharyngitis, like croup in diphtheria is a secondary affection, the characteristics of the principal disease distinguishing the local affection. The violent general symptoms, the eruption, heat of the skin, a remarkable acceleration of the pulse and the desquamation will guide the practitioner. But the differences in the local phenomena are very decided. According to Bretonneau "scarlatina anginosa is as different from diphtheria as scarlatina itself is from small pox. In the pharyngitis of scarlatina the tonsils are rather coated (enduites) by the exudation than covered (recouvertes) by membraniform filaments. The exudation in the pharyngitis of scarlatina is preceded by a *deep red color* of the whole of the pharyngial mucous membrane; in diphtheria the color of this membrane is simply inflammatory. The exudation in the former is white, opaque, and caseous, and is easily indented. The diphtheritic false membrane is grayish and so tenaceous, that it will not easily receive the impression from a hard body. Instead of commencing upon the tonsils and spreading from these to other parts, as in diphtheritic pharyngitis, the pharyngitis of scarlatina invades simultaneously the whole cavity of the fauces and nares. Lastly, the most important fact is, that pultaceous pharyngitis has not like true diphtheritic, a tendency to invade the respiratory passages, but on the contrary its extension is towards the asophagus.

The development of the false membrane upon the skin distinguishes the diphtheritic from scarlatinal pharyngitis. "Huxham and Fothergill have not spoken on this symptom, although they have described scarlatina anginosa with great care. M. Trousseau himself, does not mention it in his description of the latter disease, yet he has cited a great number of cases observed in epidemics of diphtheria."—(Valleix.)

The present epidemic furnishes two facts worthy of notice: First, in none of the 133 cases was the scarlatinal eruption observed, although searched for; Second, 34 of these cases had had scarlet fever.

*Gangrene.*—"In comparing" says M. Bretonneau, "the morbid appearances found on dissection in fifty-five subjects of all ages, who in the course of two years fell victims to the epidemic angina, in no case, even of the most malignant nature, was there anything like gangrene of the parts. Ecchymoses of small extent, and an occasional slight erosion of those surfaces where the disease had longest continued, were the gravest alterations of structure which were seen."

Although the fact that the mucous surface sub-jacent to the false membrane, preserving its integrity, is a very strong and important distinguishing characteristic of diphtheritis, yet there are exceptional cases where the diphtheritic inflammation terminates in ulceration and gangrene, the latter condition extremely rare. We have given instances of both of these terminations. We know that the color of the false membrane and its odor in some cases of diphtheritis, with a tumefaction of the surrounding mucous membrane, may so closely resemble a circumscribed gangrene that no less an acute observer than M. Bretonneau was deceived in one or two cases, but in the instance we have given there was no possibility of error, as will be seen by referring to the case.

Of the existence of gangrene of the pharynx as a primitive affection, there is no dispute, but that it ever appears in an epidemic form there is much doubt. It almost always occurs during the course of some other disease, as measles, scarlet fever, small pox, &c.

In those cases ef diphtheria which *simulate* gangrene, the manner of invasion, and t ie march of the disease, and on removal of the false membrane, the condition of the subjacent mucous surface, will clear the diagnosis.

We had intended to examine the different theories as to the origin of diphtheria, and to give a history of the disease as it has appeared in the states, as far as we were able, but we must defer these for a future occasion. We will conclude with the following quotation, as we could not express our conclusions in clearer language.

"I. Diphtheria is a *specific* disease. This is seen in its origin, march, and mode of extension ; in the character of its exudation, in its local manifestation ; in its seat of predilection ; in its *toxic* influence ; in its prodromata ; its manner of termination and its sequences.

II. It is often confounded with scarlatinal angina, and with gangrenous cynanche," and with croup, we have endeavored to indicate the diagnosis.

"III. It is propagated by infection and by contagion.

IV. The treatment should include the local application of a solution of nitrate of silver, hydrochloric acid, or the tincture of sesquichloride of iron, The internal remedies most useful are, the tincture sesquichloride of iron, hydrochloric acid, and chlorate of potash.

Nourishing diet and stimulants.

V. The means of prevention, besides careful hygienic measures—as ventilation, etc.—must also include the daily examination of the throat where the epidemie type presides, a matter of the greatest importance, as experience has fully shown, and the early isolation of the patient as soon as attacked—a precaution hardly less necessary."—*London Lancet*, May, 1859.

